# Orally delivered biodegradable targeted inflammation resolving pectin-coated nanoparticles induce anastomotic healing post intestinal surgery

**DOI:** 10.1038/s41598-024-80886-1

**Published:** 2024-11-25

**Authors:** Jong Hyun Lee, Stefan Reischl, Robert Leon Walter, Vincent Vieregge, Marie-Christin Weber, Runxin Xu, Hao Chen, Kamacay Cira, Atsuko Kasajima, Helmut Friess, Philipp-Alexander Neumann, Nazila Kamaly

**Affiliations:** 1 Department of Chemistry, Molecular Sciences Research Hub, Imperial College London, London, UK; 2 Department of Health Technology, Technical University of Denmark, Kgs. Lyngby, Denmark; 3 School of Medicine, Department of Surgery, Technical University of Munich, Munich, Germany; 4 Institute of Diagnostic and Interventional Radiology, Technical University of Munich, Munich, Germany; 5 Institute of Pathology, Technical University of Munich, Munich, Germany

**Keywords:** Anastomosis, Intestinal surgery, Inflammatory bowel diseases (IBD), Inflammation, Resolution of inflammation, Annexin A1, Ac2-26, Collagen IV, Nanomedicine, Pectin, Chitosan, Controlled release, Crohn's disease, Ulcerative colitis, Drug delivery

## Abstract

Targeted perioperative therapeutics supporting anastomotic healing during colitis are an urgent medical need. This study aimed to develop and evaluate a novel nanoparticle (NP)-based drug delivery system for improving anastomotic healing in Inflammatory bowel disease (IBD) patients following surgery. We developed pectin-coated polymeric NPs encapsulating the inflammation-resolving peptide Ac2-26. These NPs are designed to survive gastric passage, facilitate localized release in the colon via microbial pectinase degradation, and bind to the intestinal wound through collagen IV targeting. We investigated these NPs in a murine surgical model combining intestinal anastomosis with preoperative colitis induction. Perioperative administration of pectin-chitosan coated NPs containing Ac2-26 (P-C-Col IV-Ac2-26-NP) reduced colitis activity postoperatively. Macroscopic wound closure improved, as evaluated by endoscopy and intraabdominal adhesion scoring. Microscopic analysis revealed an improved semiquantitative healing score in the treatment group. This proof-of-concept study demonstrates that novel P-C-Col IV-Ac2-26-NP could be a promising and clinically feasible perioperative treatment strategy for IBD patients undergoing intestinal surgery. The targeted delivery system shows potential for enhancing anastomotic healing and reducing postoperative complications in this IBD patient population.

**Supplementary Information:**

The online version contains supplementary material available at 10.1038/s41598-024-80886-1.

## Introduction

Inflammatory bowel diseases (IBD) - comprising ulcerative colitis and Crohn´s disease - represent chronic inflammatory disorders of the gastrointestinal tract that are associated with relapsing symptoms of severe diarrhea, fever, weight loss, and abdominal pain^1,2^. Immunosuppressive medical therapy is considered the first line approach for treatment of IBD. Although medical therapy is successful in most IBD patients, over 40% require surgical interventions at least once in their lifetime^1,2^. Surgical anastomoses to reconnect the bowel after resection of an intestinal segment are frequent procedures used in IBD surgery. Despite technical advances in surgery, postoperative anastomotic leakage occurs in up to 20% of colorectal operations and leads to high morbidity^3^. Intestinal inflammation as seen in these patients can disturb the healing process, often leading to failed anastomotic healing.

The attempt to dampen the inflammation caused by active colitis prior to surgery by immunosuppressive drugs such as glucocorticoids or TNF-alpha inhibitors might even further disturb a regulated healing process after anastomosis formation. This is because throughout the anastomotic healing process, a precise balance of pro- and anti-inflammatory signaling is essential for successful healing. The initial inflammatory reaction seems to be indispensable, as treatment with non-steroidal anti-inflammatory drugs strongly reduces the inflammatory response, leading to an increase in anastomotic leakage rates^4^.

The resolution of inflammation is innately mediated by various lipids and proteins, which block inflammatory cell influx, promote their egress, clear pathogens, cellular debris and inflammatory cytokines, and repair tissue damage^5,6^. Resolution mediators include endogenous lipids that are generated during inflammation, e.g. lipoxins, resolvins, protectins, maresins; and proteins such as Annexin A1 (ANXA1), which is a key protein that is capable of facilitating resolution of inflammation^7,8^. This protein binds to the formyl peptide receptor expressed on activated cells such as phagocytes and epithelial cells and can resolve inflammation. It exerts inflammation resolution by suppression of pro-inflammatory signaling (e.g. NF-κB signaling)^9,10^. It has shown pro-resolving properties in various inflammation models that include endotoxemia, peritonitis and arthritis^11–13^. Furthermore, intraperitoneal injections of recombinant ANXA1 lead to closure of mucosal wounds induced with biopsy forceps and a reduction of colitis activity induced by dextran sodium sulfate^14^. These results make ANXA1 and its pharmacophore, the 26 amino acid *N*terminal peptide Ac2-26^15–17^ a promising candidate for perioperative treatment of IBD patients.

Although oral administration would be a cost-effective route to locally deliver biological therapeutics to influence the healing process of intestinal wounds, degradation during gastric passage complicates this strategy. Nanoparticle (NP) drug delivery systems allow encapsulation of the therapeutic agent, and can protect biological payloads during gastric passage if gut resistant compositions are used. Amongst the most successfully translated nanomedicines are drug loaded polymeric NPs, which have proven to be promising tools for the specific and sustained delivery of a range of therapeutics^18–23^. Polymeric NPs are also currently being investigated for oral delivery applications^24^^,25^.NP-mediated delivery of biologics, including resolution mediators, can facilitate their: (a) targeted delivery; (b) controlled release, which facilitates dose tempering; and (c) protection from stomach components until release at target site. Furthermore, pectin-chitosan coating of polymeric NPs allows for protection against degradation during gastric passage with localized release occurring after specific pectin digestion in the colon by microbial pectinases and colonic bacteria^26–28^. We have previously shown that intraperitoneal application of polymeric Ac2-26-NPs is effective in inducing intestinal wound healing processes and recovery from experimental colitis^29–31^. These results suggest a high therapeutic potential for NP encapsulated Ac2-26 to induce anastomotic healing during colitis.

**Fig. 1:**
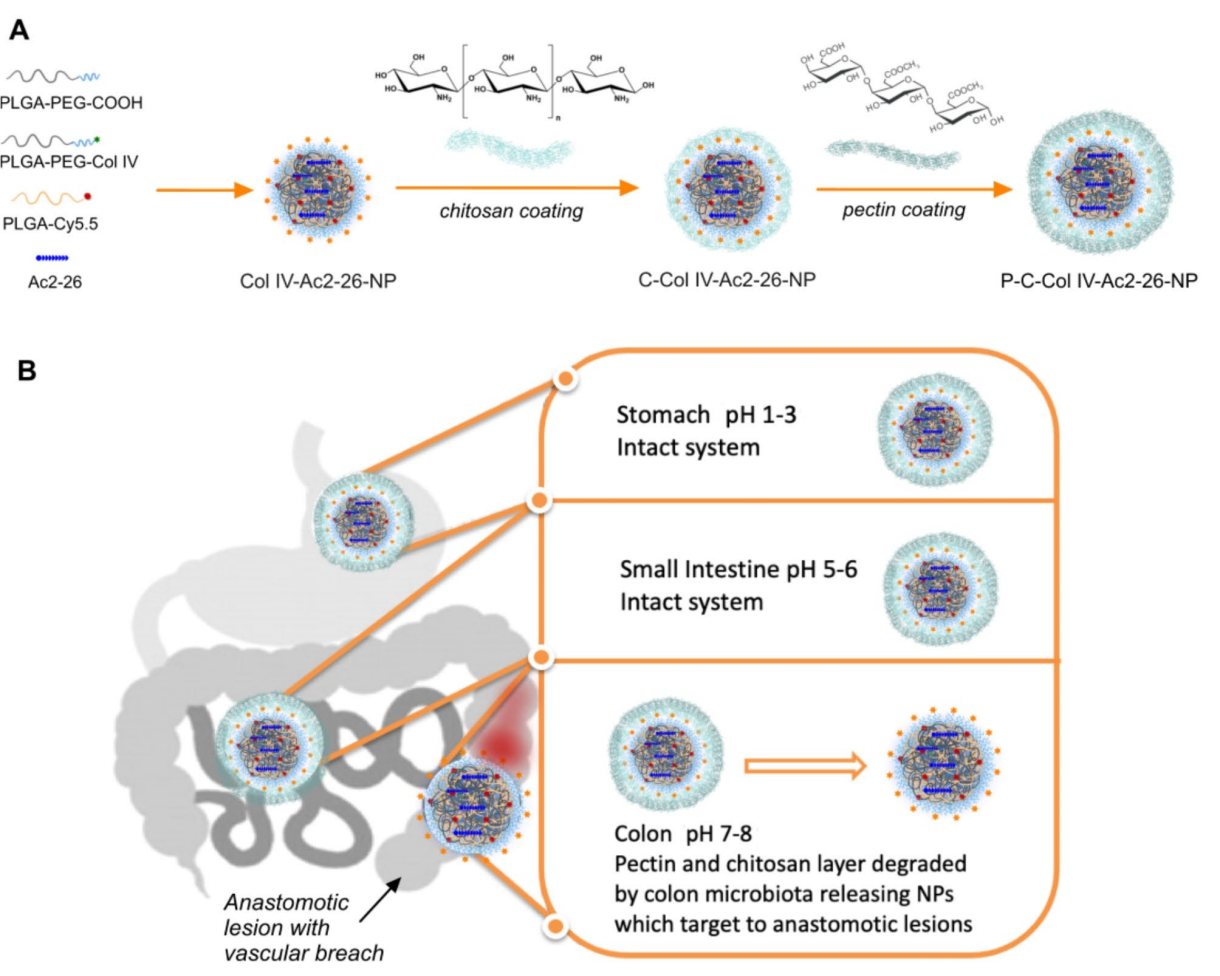
Overall design of biodegradable layer-by-layer P-C-Col IV Ac2-26 NPs for anastomotic healing. The pectin coating of the NPs protects from the harsh pH environment of the stomach, small intestine and enzymes secreted for digestion. Once in the colon, pectin and the subsequent chitosan layers are fermented and degraded almost completely by the microflora in the gut and this leads to the local release of the Col-IV Ac2-26 NPs, which binds to wounds and releases the Ac2-26 peptide in situ.

In this current work, we aimed to further develop these NPs for oral administration by coating them initially with a chitosan layer, followed by a subsequent pectin layer for degradation within the colon and release of the core collagen IV targerted Ac2-26 NPs (Fig. 1A). The orally administered NPs have to withstand various environmental conditions in the gastrointestinal tract (GIT), a low pH in the stomach, mechanical pressure, protease attack in the small intestine and microflora digestion in the colon. In order to minimize the impact on the stability of the Ac2-26 peptide and the targeting collagen IV peptide on the surface of the NPs, following a chitosan coating, we further coated the NPs with pectin, which is a naturally occurring edible polysaccharide with a long and safe history of use in the food industry^32^. In fact, pectin passes intact through the upper GI tract and is degraded by colonic microflora, which will ensure degradation and NP release at the anastomotic site within the colon. Pectin on its own cannot be used to deliver the naked Ac2-26 peptide, as pectin swells considerably in physiological conditions and the peptide will be released prematurely through the large pores. We therefore encapsulated polylactic-co-glycolic acid (PLGA) NPs entrapping the Ac2-26 peptides with a short PEGylated surface and collagen IV targeting peptides within chitosan and subsequent pectin layers and tested these in anastomosis models. The additional chitosan and pectin layers help protect the collagen IV targeting peptide ligand, in addition to the Ac2-26 peptide until the NPs have reached the lower colon where peptinases and colon microbiota can degrade their pectin and chitosan layers, releasing the Col IV-Ac2-26 NP to target the anasotomotic site (Fig. 1B).

## Results and discussion

### Development of pectin-coated polymeric Ac2-26 loaded nanoparticles (P-C-Col IV-Ac2-26 NPs)

We have previously shown that collagen-IV-targeted Ac2-26 containing nanoparticles (Col IV-Ac2-26-NPs) are inflammation resolving in murine peritonitis, hind-limb reperfusion injury, advanced atherosclerosis, mucosal wound repair and experimental colitis^10,20,29,30^. In this study, we developed a modified version of these NPs with a chitosan and pectin coating for oral delivery and investigated their efficacy in our anastomotic healing model during colitis. Most importantly, the use of controlled release polymeric NPs can facilitate dose tempering of the potent pro-resolving Ac2-26 peptide, which suffers from short plasma-half life and non-specific effects when administered in its free form. While our previous studies demonstrated the efficacy of Ac2-26 loaded PLGA NPs with collagen IV targeting in peritonitis and intestinal inflammation models, the current study introduces a novel oral delivery approach for these NPs. We focused on developing a system that could withstand the harsh conditions of the gastrointestinal tract and provide targeted delivery to the colon. To achieve this, we coated the NPs with chitosan and pectin. Pectin was chosen as the outer coating due to its unique properties that make it ideal for colon-targeted delivery. Unlike enteric-coated polymers that rely on pH changes, pectin remains intact in the upper GI tract but is specifically degraded by colonic microflora. This approach ensures that the NPs reach the colon intact, where the pectin and chitosan layers are degraded, exposing the collagen IV-targeted Ac2-26 NPs. This novel delivery system combines the benefits of our previously studied NPs with a colon-specific release mechanism, improving the efficacy and feasibility of perioperative treatment for IBD patients undergoing intestinal surgery.

In the first instance we synthesised the required copolymers of poly[lactic-*co*-glycolic acid]-*b*-poly[ethylene glycol] (PLGA-PEG) and PLGA-PEG-Col IV as previously reported^30^. The major feature of these polymers is that they utilise a very short PEG sequence of about 534.6 Da in order to allow for chitosan coating, which provides the positive electrostatic interaction with the negatively charged pectin polymer – both chitosan and pectin are degradable once the NPs have reached the colon. For these NPs, a collagen IV (Col IV) targeting approach was used to retain the Ac2-26-NPs (post chitosan and pectin biodegradation) at inflamed or wounded intestinal tissue and to enable the release of the Ac2-26 peptide. Col IV is a major component of the basal membrane, and is exposed at sites of tissue injury, hence serving as an optimal extracellular matrix targeting strategy^33^.

**Fig. 2:**
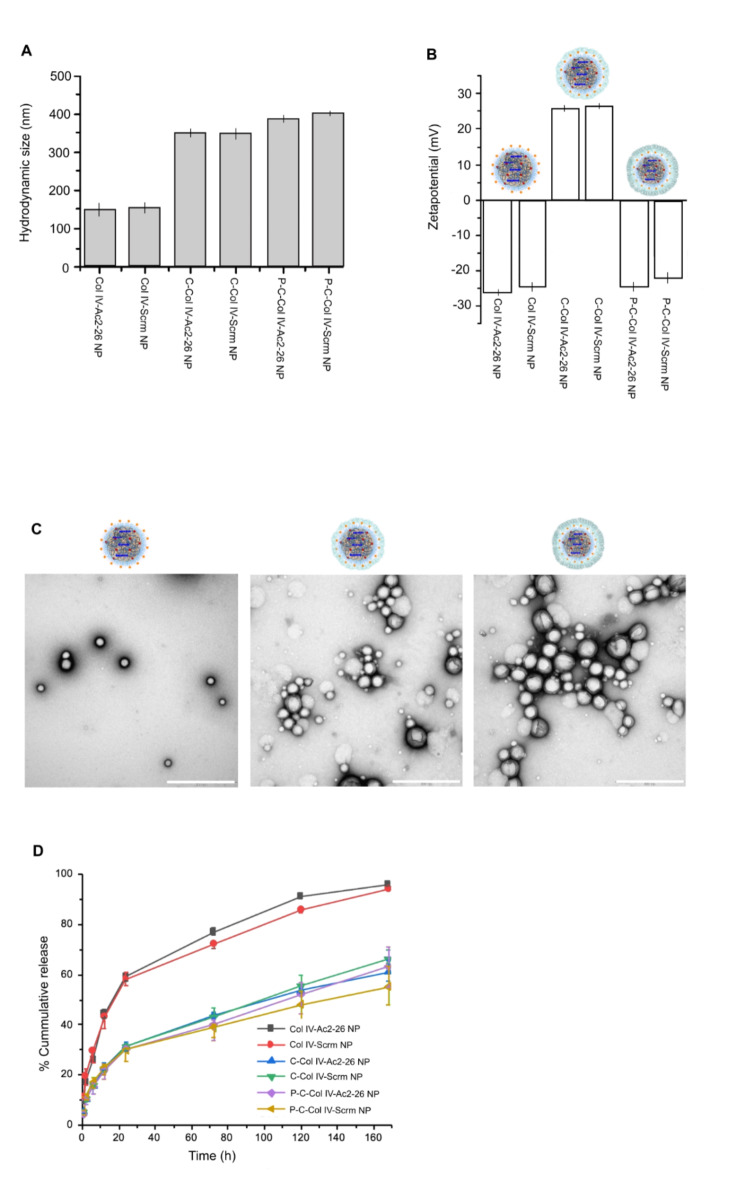
Biodegradable layer-by-layer P-C-Col IV Ac2-26 NP characterisation. Collagen IV-targeted (Col-IV) NPs encapsulating the Ac2-26 peptide (Ac2-26-NP) or controls including a randomly generated, isoelectric mismatched scrambled sequence (Scrm-NP), were developed using biodegradable polymers via a single-step nanoprecipitation method, C: chitosan, P: pectin. (A) Hydrodynamic size of empty (NP), Ac2-26 loaded (Ac2-26-NP) and scrambled peptide (Scrm-NP) NPs were measured (mean ± SD, *n* = 3). (B) ζ-Potential of the NPs indicating surface charge in PBS at pH 7.4. (C) Representative TEM image of Col IV-Ac2-26-NPs (left) C-Col IV-Ac2-26 NPs (middle) and P-C-Col IV-Ac2-26 NPs (right) (scale bar in white = 500 nm) (D) In vitro cumulative release curve of Ac2-26 or scrambled peptide from NPs in PBS incubated at 37 °C is shown (mean ± SD, *n* = 3).

In addition to Col IV-targeted Ac2-26 NPs, targeted NPs containing a randomly generated, isoelectric miss-matched scrambled peptide sequence to Ac2-26 (Scrm-NP) were also formulated as controls. The NP encapsulation efficiency for the Ac2-26 peptide and scrambled peptide were 87.5 and 91.7%, respectively. The loading efficiency for the Ac2-26 peptide and scrambled peptide were 10.5 and 11%, respectively. Col IV Ac2-26 NPs and Col IV Scrm NPs had a hydrodynamic size distribution between 149.0 ± 2.5 nm and 155.25 ± 3.7 nm, indicating these two NPs are close in size for effective comparison (Fig. 2A). We then observed an increase in hydrodynamic size for these NPs following electrostatic coating with chitosan up to approximately 350 nm, indicating extra layers existing on the surface of the NPs following this intial coating (Fig. 2A). Next, the NPs were electrostatically coated with pectin and in this case their hydrodynamic size increased up to 400 nm, further indicating electrostatic binding of the pectin polymer to the surface of the NPs. The surface charge (ζ-potential) of the NPs in PBS at pH 7.4 was measured to be as follows: Col IV-Ac2-26 NP (−26.33 mV ± 0.86), Col IV-Scrm NP (−24.8 mV ± 1.4), C-Col IV-Ac2-26 NP (26.6 mV ± 0.9), C-Col IV-Scrm NP (27 mV ± 0.5), P-C-Col IV-Ac2-26 NP (−25.6 mV ± 3.1), P-C-Col IV-Scrm (−23.5 mV ± 1.5), (Fig. 2B). The spherical shape and morphology of the NPs was confirmed with TEM, and the extra coated layers were identifiable as a darker shell layer around the NPs (Fig. 2C). The release profiles of Ac2-26 and the scrambled peptide from the nanoparticles, as shown in Fig. 2D, demonstrate the impact of the chitosan and pectin coatings on the release kinetics. The uncoated NPs exhibited the fastest release rate, with approximately 60% of the peptide released within the first 24 h, and reaching nearly 80% release by 72 h. In contrast, the chitosan-coated NPs (C-Col IV-Ac2-26 NP) showed a more controlled release profile, with about 30% release at 24 h and approximately 40% by 72 h. The pectin-chitosan coated NPs (P-C-Col IV-Ac2-26 NP) demonstrated the most sustained release, with only about 20% of the peptide released at 24 h and approximately 38% by 72 h. This gradual release from the P-C-Col IV-Ac2-26 NPs continued over the 7-day period, reaching about 60% cumulative release. The similar release profiles observed for both Ac2-26 and the scrambled peptide suggest that the release kinetics are primarily governed by the NP structure and coatings rather than the specific peptide properties. These results indicate that the pectin-chitosan coating effectively delays peptide release, which is crucial for protecting the payload during transit through the upper gastrointestinal tract and allowing for targeted release in the colon.

## Oral administration of P-C-Col IV-Ac2-26 NP tempers postoperative colitis activity

**Fig. 3:**
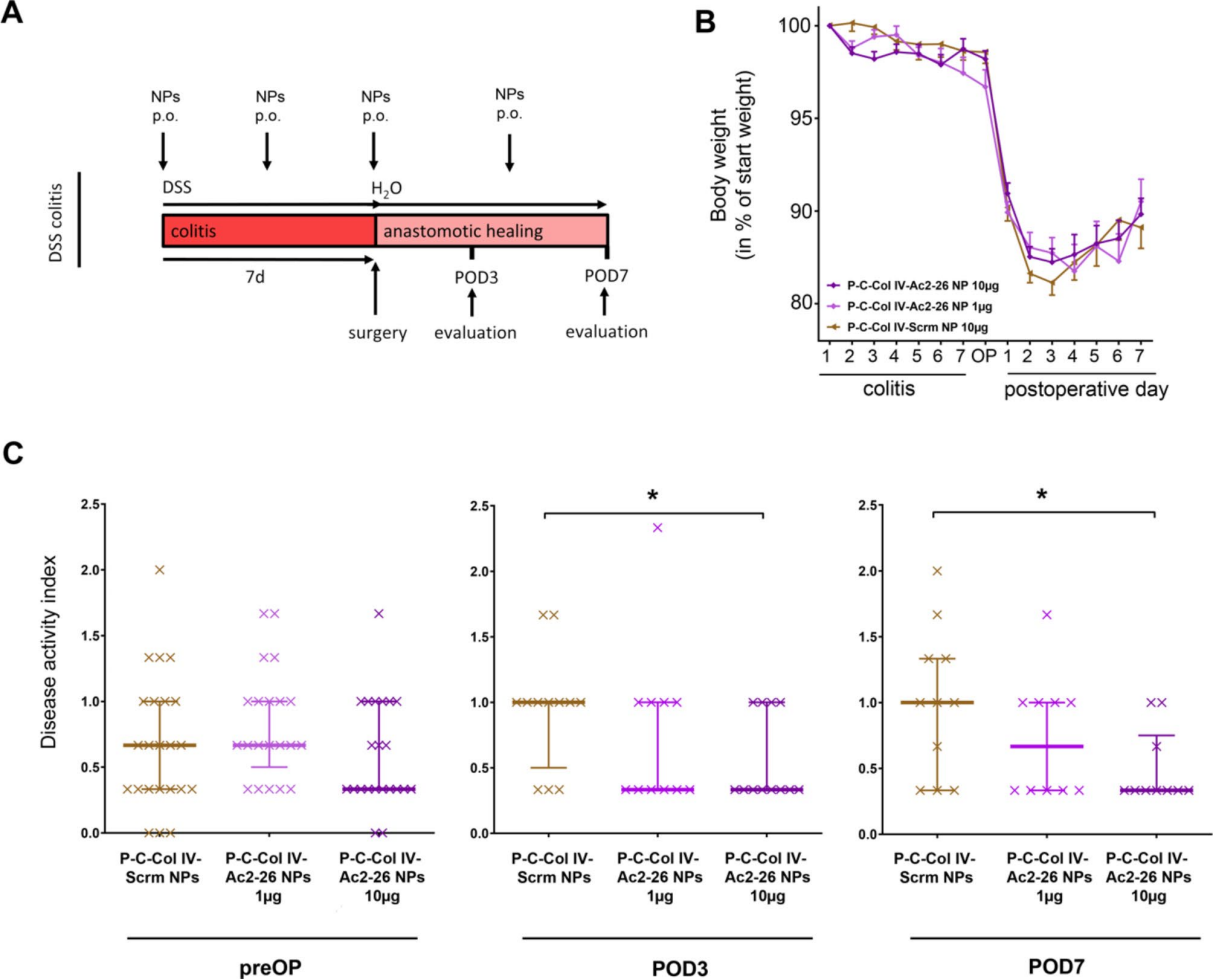
Perioperative colitis disease activity is improved by oral treatment with pectin-chitosan coated Col IV Ac2-26-nanoparticles (P-C-Col IV-Ac2-26 NPs). (A) Timeline of the experimental procedure. Mice were treated by 2% DSS in drinking water. After 7 days DSS was changed to normal drinking water and microsurgical colonic anastomosis was performed. Mice were treated every 3.5 days with oral gavage of pectin-coated P-C-Col IV-Ac2-26 NPs in different doses (1 µg Ac2-26–10 µg Ac2-26) or pectin-coated Scrambled-nanoparticles (P-C-Col IV-Scrm NPs at a dose of 10 µg of Scrm peptide) throughout the whole procedure. (B) Body weight is shown as percentage of starting weight every day during the whole procedure. (C) Disease activity index (DAI) at 3 different time points is shown: immediately preoperatively (preOP), at postoperative day 3 (POD3) and at postoperative day 7 (POD7). Statistical differences determined by Mann-Whitney-U test for nonparametric results are indicated with *(*P* < 0.05). *N* = 23 (P-C-Col IV-Scrm NPs), *N* = 21 (P-C-Col IV-Ac2-26 NPs 1 µg), *N* = 22 (P-C-Col IV-Ac2-26 NPs 10 µg). Three animals were excluded from analyses for reaching abort criteria (1 per each treatment group), 3 animals were excluded for intraoperative technical problems (2 in P-C-Col IV-Ac2-26 NPs 1 µg, 1 in P-C-Col IV-Ac2-26 NPs 10 µg).

In our murine surgical IBD model, we combined a 7 day preoperative phase of experimental DSS colitis with the surgical formation of a colon anastomosis in microsurgical technique. DSS administration led to pronounced induction of colitis, which was confirmed histopathologically in whole colonic slices (supplemental Fig. 1). Here, the P-C-Col IV Ac2-26-NP or P-C-Col IV Scrm-NP as control were administered through regular oral gavage every 3.5 days, starting with induction of colitis (Fig. 3A). This therapeutic scheme seems appropriate, as in the clinical setting the administration of the treatment can be initiated as soon as the indication for surgery is made during acute colitis.

Mice receiving oral administrations of 10 µg P-C-Col IV-Ac2-26 NP had a significantly improved disease activity index (DAI) at POD 3 (median 1 vs. 0.3, *p* < 0.02) and POD 7 (median 1 vs. 0.3, *p* < 0.02) compared to Scrm-NP treated mice. At the lower dose of 1 µg P-C-Col IV-Ac2-26NP there was an improvement of the DAI at POD3 and POD7, although this was not significant. Immediately before surgery, the DAI did not significantly differ between either of the treatment groups (Fig. 3C). Body weight was measured as a marker of postoperative recovery, but did not differ between the treatment groups (Fig. 3B).

## P-C-Col IV Ac2-26-NPs induce anastomotic wound closure and intestinal leakage

**Fig. 4:**
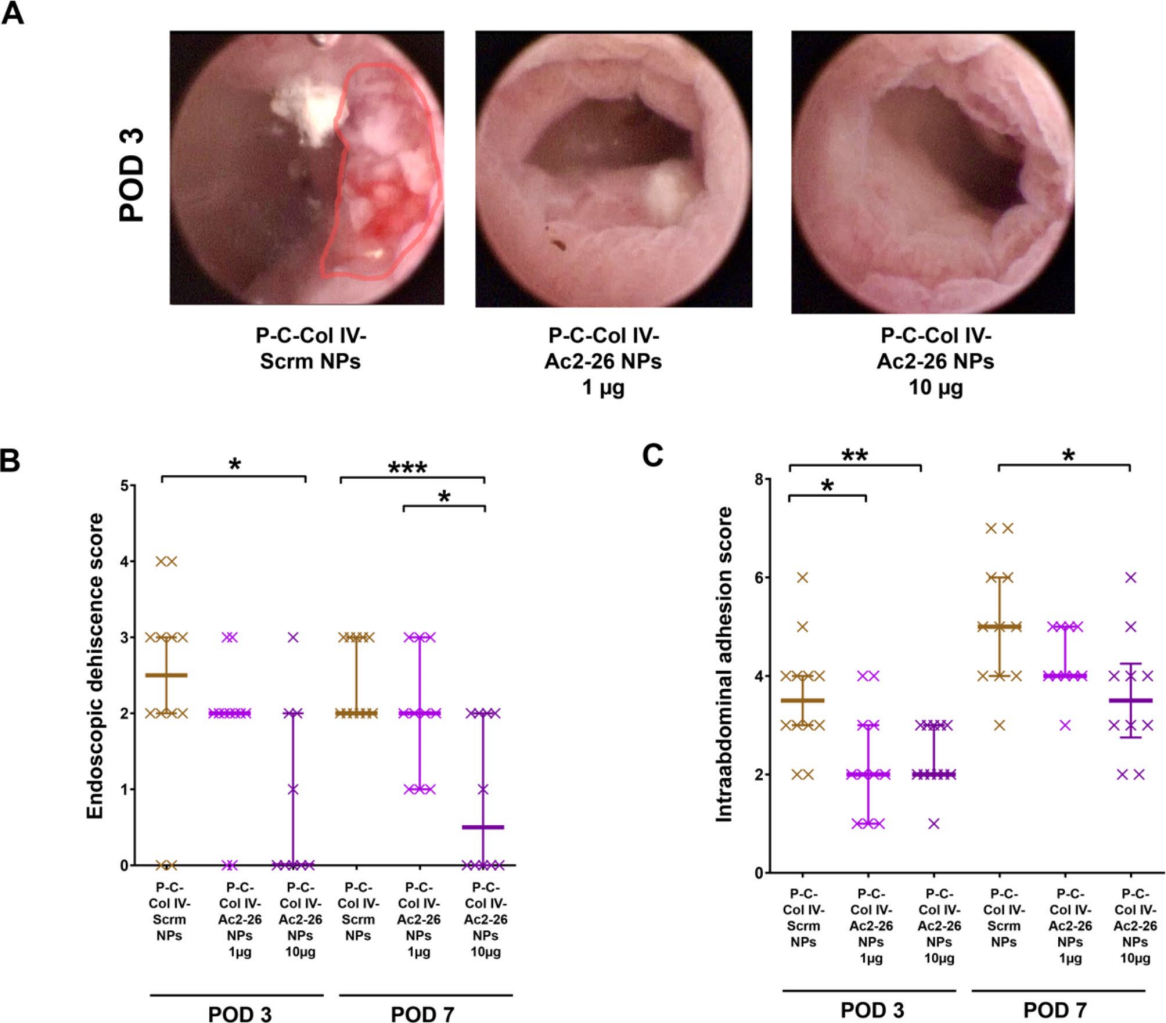
Macroscopic wound closure is improved by by oral treatment with P-C-Col IV-Ac2-26 NP. (A) Rigid colonic endoscopy was performed to analyze luminal macroscopic wound closure. Representative anastomoses at postoperative day 3 are shown. Dehiscent areas are marked in red. (B) Endoscopic deshiscence score evaluates local wound healing from a luminal perspective. Anastomotic healing was scored as described before34: no dehiscence = 0; sutures protruding into the lumen = 1; dehiscence less than 25% of circumference = 2; dehiscence more than 25% of circumference = 3; complete dehiscence = 4. (C) Anastomotic leakage leads to adhesion formation with the adjacent tissue. Therefore grade of anastomotic leakage could be measured as follows: adhesion of mesenteric fat tissue, uterus, intestine or other organs was awarded with 1 point each. Abundance of adhesions was scored according to the following score and added: no adhesion = 0; completely bluntly removable = 1; partially bluntly removable = 2; not bluntly removable = 3. Statistical differences determined by Mann Whitney U test for nonparametric results are indicated with *(*P* < 0.05), **(*P* < 0.01), ***(*P* < 0.001). *N* = 23 (P-C-Col IV-Scrm NPs), *N* = 21 (P-C-Col IV-Ac2-26 NPs 1 µg), *N* = 22 (P-C-Col IV-Ac2-26 NPs 10 µg). Three animals were excluded from analyses for reaching abort criteria (1 per each treatment group), 3 animals were excluded for intraoperative technical problems (2 in P-CCol IV-Ac2-26 NPs 1 µg, 1 in P-C-Col IV-Ac2-26 NPs 10 µg), while additionally 3 were removed from analysis in P-C-Col IV-Ac2-26 NPs 10 µg at POD 3 for technical endoscopy problems.

Wound closure was evaluated from the luminal side and from the abdominal side. Rigid endoscopy revealed significantly lower deciscence scores in the mice treated with 10 µg Ac2-26-pNP compared to P-C-Col IV Scrm-NPs at POD 3 (median 0 vs. 2.5, *p* < 0.02) and POD 7 (median 0.5 vs. 2, *p* < 0.0002) (Fig. 4, A-B). Scores of the mice treated with 1 µg of P-C-Col IV Ac2-26-NPs were in between these groups at POD 3 and 7 demonstrating a dose dependency of the effect. Leakage from the wound causes intraabdominal inflammatory collections adjacent to the anastomosis, which lead to adhesions of the adjacent intraabdominal organs and fat tissue. Thereby adhesion formation reflects the degree of leakage during the healing process. We could observe significantly reduced adhesion scores at POD 3 (median 2 vs. 3.7, *p* < 0.004) and POD 7 (3.7 vs. 5, *p* < 0.02) in the mice treated with 10 µg of P-C-Col IV Ac2-26-NPs compared to Scrm-NP (Fig. 4C). Again, this effect was dose dependent.

*Orally administered P-C-Col IV-Ac2-26 NP promote the healing process in histological analysis.*

**Fig. 5:**
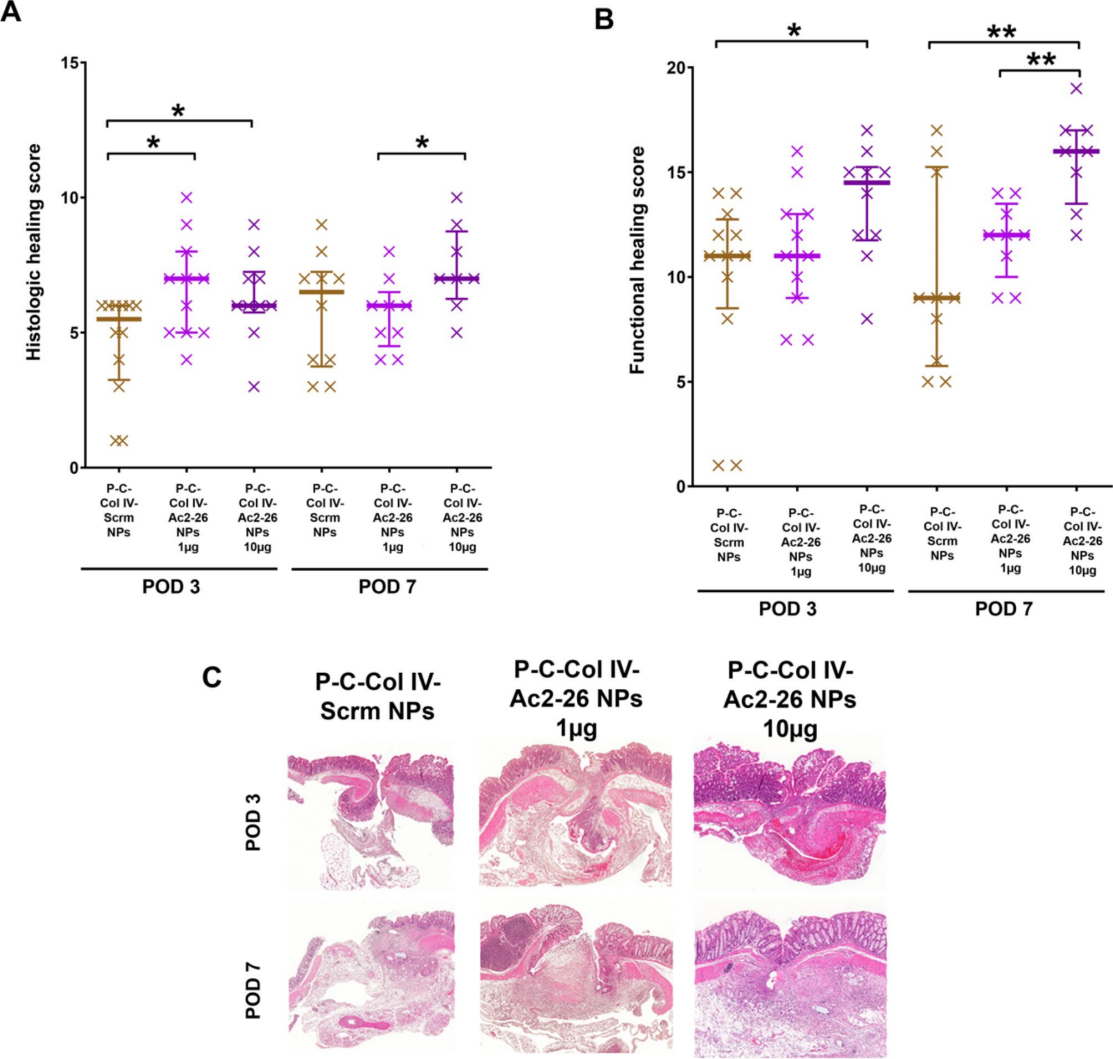
Microscopic intestinal wound healing is improved by perioperative oral administation with P-C-Col IV-Ac2-26 NPs.(A) HE-stained longitudinal sections of the anastomosis were analyzed by an experienced pathologist according to the established Phillips-Score. A score of 0 (not detectable) – 4 (highly detectable) was awarded to the following items and summed up: blood vessel ingrowth, fibroblast ingrowth, collagen formation and absence of inflammatory cells. (B) Microscopical wound closure was scored by adding additional items to the Phillips-score, focusing on closure of the individual intestinal layers^34^. (C) Representative HE-stained sections of the different groups are shown. Statistical differences determined by Mann Whitney U test for nonparametric results are indicated with *(*P* < 0.05), **(*P* < 0.01). *N* = 23 (P-C-Col IV-Scrm NPs), *N* = 21 (P-C-Col IV-Ac2-26 NPs 1 µg), *N* = 22 (P-C-Col IV-Ac2-26 NPs 10 µg). Three animals were excluded from analyses for reaching abort criteria (1 per each treatment group), 3 animals were excluded for intraoperative technical problems (2 in P-C-Col IV-Ac2-26 NPs 1 µg, 1 in P-C-Col IV-Ac2-26 NPs 10 µg).

Additional to macroscopic evaluation of wound closure, microscopic analysis was performed to analyse the effects on the healing process at the histological level. The well-established healing score published by Phillips et al. was used for quantification of the cornerstones of regular healing: resolution of the inflammatory response, fibroblast ingrowth and subsequent collagen formation and angiogenesis^35^. At POD 3, th1§e healing score was higher in both doses of P-C-Col IV-Ac2-26 NP compared to the P-C-Col IV-Scrm NP control group (median 6.5 for 1 µg P-C-Col IV-Ac2-26 NP, 6 for 10 µg P-C-Col IV-Ac2-26 NP vs. 5.5 for P-C-Col IV-Scrm NP) (Fig. 5, A-C). At POD 7, there was no significant difference between P-C-Col IV-Scrm NP treated animals and each of the P-C-Col IV-Ac2-26NP treatment groups. These results are indicating the effect of P-C-Col IV-Ac2-26 NP especially in the early healing phase. In the functional healing score, which was adding more items including scoring the closure of the individual bowel wall layers, treatment with 10 µg and 1 µg P-C-Col IV-Ac2-26 NP significantly improved healing at POD 7 compared to control (Fig. 5, B-C). At POD3 only the high dose of 10 µg of P-C-Col IV-Ac2-26NP improved wound healing compared to the placebo group, but not the dose of 1 µg of Ac2-26.

## Conclusion

In summary, proresolving treatment with pectin coated collagen-IV-targeted Ac2-26-loaded NPs represents a novel perioperative treatment strategy with a high translational potential for patients with IBD required surgery. This approach is particularly promising additionally to established anti-inflammatory medications in the perioperative phase, as oral administration is clinically feasible, allows for gastric passage and mediates significant improvement of intestinal anastomotic healing and colitis activity.

## Methods

*Materials and reagents*.

The biodegradable Poly(DL-lactic-co-glycolic acid) (50:50) (PLGA, MW: kDa), heterobifunctional NH_2_-PEG-COOH (MW: 534.6 Da) and OH-PEG-maleimide (MW: 617 Da) polymers were purchased from Nanosoft Polymers. Ac2-26 peptide (AMVSEFLKQAWFIENEEQEYVQTVK) was purchased from Tocris Biosciences. The Collagen IV targeting peptide (KLWVLPKGGGC) and the scrambled Ac2-26 peptide (WLKQKFQESVEQIAYVMENATEFEV) were purchased from Mimotopes. All NPs included a 5 molar % of PLGA.Cy5.5 fluorescent polymer for tracking studies. All methods were performed in accordance with the relevant guidelines and regulations of both Imperial College London and the Technical University of Munich. All animal procedures were approved by the local animal welfare committee of the regional administration of Upper Bavaria, Germany (Protocol No. 55.2–2532.Vet_02-17–203).

*Synthesis of NPs*.

PLGA-PEG-COOH, PLGA-PEG-Col IV and PLGA-PEG-Cy5.5 were synthesised as previously reported^30^. Ac2-26 or scrambled peptide loaded PLGA NPs were prepared by a single-step nanoprecipitation self-assembly method. Either Ac2-26 or scrambled peptide (120 µg) were added to the polymer mixture containing PLGA-PEG-COOH, PLGA-PEG-Col IV, PLGA-Cy5.5 (3 mg total polymer mass) in 1 mL acetonitrile, and the solution was gently vortexed overnight at RT. This polymer and peptide mixture was then added in a dropwise manner to 10mL of distilled water under gentle stirring and the solution stirred at RT for 4 h and filtered through sterile 0.45 μm syringe filters (regenerated cellulose, 17 mm, Cole Palmer Instruments). The NPs were concentrated by centrifugation at 3000 x g for 20 min using Amicon Ultra-15 centrifugal filter units (MWCO 100KDa, Millipore Ltd), washed with deionized water, and resuspended in 1mL of H_2_O, and then further diluted with PBS prior to injection at a concentration of 1 µg Ac2-26. This solution was then dropwise added to a 1% wt ratio chitosan solution. The solution was stirred overnight and filtered through sterile 0.45 μm syringe filters (regenerated cellulose, 17 mm, Cole Palmer Instruments). The chitosan coated NPs were concentrated by centrifugation at 3000 x g for 20 min using Amicon Ultra-15 centrifugal filter units (MWCO 100KDa, Sigma-Aldrich), washed with deionized water, and resuspended in 1 mL of nuclease free H_2_O (total 3.15 mg/mL chitosan coated NP). For pectin coating the chitosan coated NPs were added dropwise to a 1 wt% solution of pectin and the NPs stirred overnight and filter the solution through sterile 0.45 μm syringe filters (regenerated cellulose, 17 mm, Cole Palmer Instruments). The pectin, chitosan coated NPs were concentrated by centrifugation at 3000 x *g* for 20 min using Amicon Ultra-15 centrifugal filter units (MWCO 100KDa, Sigma-Aldrich), washed with deionized water, and resuspended in 1 mL of nuclease free H_2_O (total 3.18 mg/mL pectin, chitosan coated NP).

### NP characterization

Ac2-26 and scrambled peptide quantification measurements were carried out on a Spark Multimode Microplate Reader. The NP mean hydrodynamic sizes, size distribution, and z-potentials were determined by Malvern Nano ZS Zetasizer at 25 ^o^C. All measurements were carried out in triplicates. The average particle hydrodynamic size was expressed in intensity mean diameter and the reported value was represented as mean ± average (*n* = 3). For TEM, a 10 µl solution of 1 mg/ml freshly prepared NPs in H_2_O was deposited on carbon coated copper grids, the excess solution was blotted, and the grids were immersed in a solution of 0.75% uranyl formate stain. The stain was blotted, and the sample imaged within 1 h of preparation on a Tecnai™ G^2^ Spirit BioTwin electron microscope equipped with an AMT 2k CCD camera and low-dose software (80 kV, direct mag. 98000x).

## Ac2-26 and Scrm-Ac2-26 release kinetics from NPs

To quantify the Ac2-26 release profile, 3.12 mg/mL NP samples (either P-C-Col IV-Ac2-26-NPs or P-C-Col IV-Scrm-NPs) were made in PBS, and the NPs were incubated at 37 °C. At defined time intervals, the NPs were removed, transferred to Amicon Ultra-15 centrifugal filter units (MWCO 10 kDa; Sigma-Aldrich), and centrifuged at 3,000 x g for 20 min. The NPs were then resuspended in PBS, and incubation at 37 ^o^C was continued until the designated time point. The filtrate (10 µL) was analyzed with a nanodrop UV-Vis spectrometer, and absorbance was measured at 220 nm to determine the amount of released peptide at each time point and a cumulative % release curve generated.

## Animal experiments and study design

Female BALB/c mice of 11–16 weeks with body weight between 19 and 24 g were used for the experiments (Charles River, Sulzfeld, Germany). The local animal facility environment had a temperature of 22 °C and a humidity of 45–60% followed a 12-hour light/dark cycle. Animals were kept at specific-pathogen free (SPF) conditions throughout the procedure. An initial period of 1 week prior to initiation of the experiments was allowed for adaption to housing conditions.

DSS was administered for 7 days in drinking water for colitis induction which causes an inflammatory reaction by permeabilization of the mucosa for bacterial influx to the lamina propria^36^. DSS was offered ad libitum in drinking water at 2% (w/v) of DSS (molecular weight 36,000–50,000, MP Biomedicals, Heidelberg, Germany). The DAI is an established score to semi quantitatively score severity of experimental colitis by scoring weight loss, blood in stolls and stool consistency was measured prior to surgery and before sacrification.

After 7 days of DSS, the surgical anastomosis was performed and postoperatively drinking water was switched to normal water. Mice were treated throughout the whole procedure by verum ( P-C-Col IV-Ac2-26 NPs, either 1–10 µg ) and placebo ( P-C-Col IV-Scrm-NP 10 µg) every 3.5 days throughout the whole procedure. Medication was administered by oral gavage by a 20G plastic cannula. Tissue was harvested at either POD 3 or 7 after endocopy and sacrification by isoflurane general anaesthesia and cervical dislocation.

### Surgical procedure

Anaesthesia was initiated by a mixture of 5% isoflurane (CP-Pharma, Burgdorf, Germany) and remainder of oxygen, and was maintained under reduced isoflurane concentrations of 1–3% to allow spontaneous breathing. Body temperature was maintained at 37 °C by a heating pad. Subcutaneous injections of meloxicam (Boehringer Ingelheim Pharma, Ingelheim, Germany) and buprenorphine (Indivior, Berkshire, UK) were used for analgesia. After laparotomy in supine position, the colon was transected at the height of the lower renal pole, preserving the vasculature. Using an operating microscope (Carl Zeiss, Oberkochen, Germany), a standardized end-to-end anastomosis was performed in microsurgical technique by 12 single stitches of monofilamentous Ethilon 9 − 0 (Ethicon, Norderstedt, Germany). Postoperatively, mice had free access to regular drinking water and food *ad. libitum*. Postoperative daily assessment included body weight measurements, observation of wound healing and administration of analgesics .

### Murine colonoscopy and dehiscence scoring

Before sacrification at POD 3 or 7, colonoscopy was performedusing the Coloview system Mainz (Karl Storz, Tuttingen, Germany) with a 7 Charriere examination shaft prior to evaluation in isoflurane sedation. Instillagel (FARCO-Pharma, Cologne, Germany) was used as lubricant.The mucosa and anastomosis were evaluated under retraction of the colonoscope. Wound healing was scored according to the following score: 0 = no dehiscence, 1 = suture protruding into the lumen, 2 = dehiscence less than 25% of the circumference, 3 = dehiscence more than 25% of the circumference, 4 = complete dehiscence, as described before^34^.

### Tissue harvesting and analysis

Mice were sacrificed by cervical dislocation under isoflurane sedation. The abdomen was opened and scoring of the adhesions was performed (Range 0–7): Adhesion of mesenterial fat, the uterus, intestine or other organs was scored with 1 point each. No adhesions was scored with 0, bluntly completely removable tissue with 1, partially removable tissue with 2 and non-removable adhesions with 3. For tissue harvesting, the descending colon was resected at least 1 cm proximally and distally from the anastomosis. Prior to histological assessment, the bowel was cut in half lengthwise. Consecutive Sect. (4 μm thickness) of the colon bearing the anastomosis were prepared and subjected to H&E staining. Anastomotic healing was in histological sections of the anastomotic area by a pathology specialist by a healing score^35^: Scores for inflammatory cells (0–4 points, this score was inversed as higher inflammatory cell numbers reflect impaired healing), angiogenesis (0–4 points), collagen synthesis (0–4 points), fibroblast ingrowth (0–4 points) were summed up. A functional score evaluating the closure of the bowel wall was performed by adding the following items^34^: first closed layer counted from extraluminal side (0 = no healed layer, 1 = serosa layer, 2 = muscular layer, 3 = submucosal layer, 4 = mucosal layer), number of healed layers (counting mucosa, submucosa, muscularis, serosa) (0–4), epithelial layer closed (0 = no, 1 = yes) and intact crypt architecture (0 = no, 1 = yes) were added for the functional healing score.

### Statistics

The results are presented as mean ± standard error of the mean (SEM) for parametric or median for non-parametric results. Unpaired two-tailed t-test or one-way analysis of variance (ANOVA) combined with Bonferroni post-test were applied to determine statistical significance for parametric data. For non-parametric data Mann-Whitney-U test were used. Data plotting and statistical analyses were performed in Prism (GraphPad, San Diego, USA).

## Electronic supplementary material

Below is the link to the electronic supplementary material.

Supplementary Material 1

Supplementary Material 2

## Data Availability

The datasets used and/or analysed during the current study available from the corresponding author on reasonable request.
