# Supplementary material for: Orally delivered biodegradable targeted inflammation resolving pectin-coated nanoparticles induce anastomotic healing post intestinal surgery
Source: Sci Rep. 2024 Nov 25;14:29253. doi: 10.1038/s41598-024-80886-1 (PMC11589105; doi:10.1038/s41598-024-80886-1)
Supplement: Supplementary file 1 — Supplementary Material 1 [file 41598_2024_80886_MOESM1_ESM.docx]

**Supplementary Information**

**Orally delivered biodegradable targeted inflammation resolving pectin-coated nanoparticles induce anastomotic healing post intestinal surgery**

Jong Hyun Lee,^1,2†^ Stefan Reischl^3,4†^, Robert Leon Walter^3^, Vincent Vieregge^3^, Marie-Christin Weber^3^, Runxin Xu,^1^ Hao Chen,^1^ Kamacay Cira^3^, Atsuko Kasajima^5^, Helmut Friess^4^, Philipp-Alexander Neumann*^3^ and Nazila Kamaly*^1^

^1^ Imperial College London, Department of Chemistry, Molecular Sciences Research Hub, United Kingdom

^2^ Technical University of Denmark, Department of Health Technology, Kgs. Lyngby, Denmark

^3^ Technical University of Munich, School of Medicine, Department of Surgery, Germany

^4^ Technical University of Munich, Institute of Diagnostic and Interventional Radiology, Germany

^5^ Technical University of Munich, Institute of Pathology, Germany

^†,^*These authors contributed equally to this work

**Correspondence:** Email: [nazila.kamaly@imperial.ac.uk](mailto:nazila.kamaly@imperial.ac.uk) and [philipp-alexander.neumann@tum.de](mailto:philipp-alexander.neumann@tum.de)


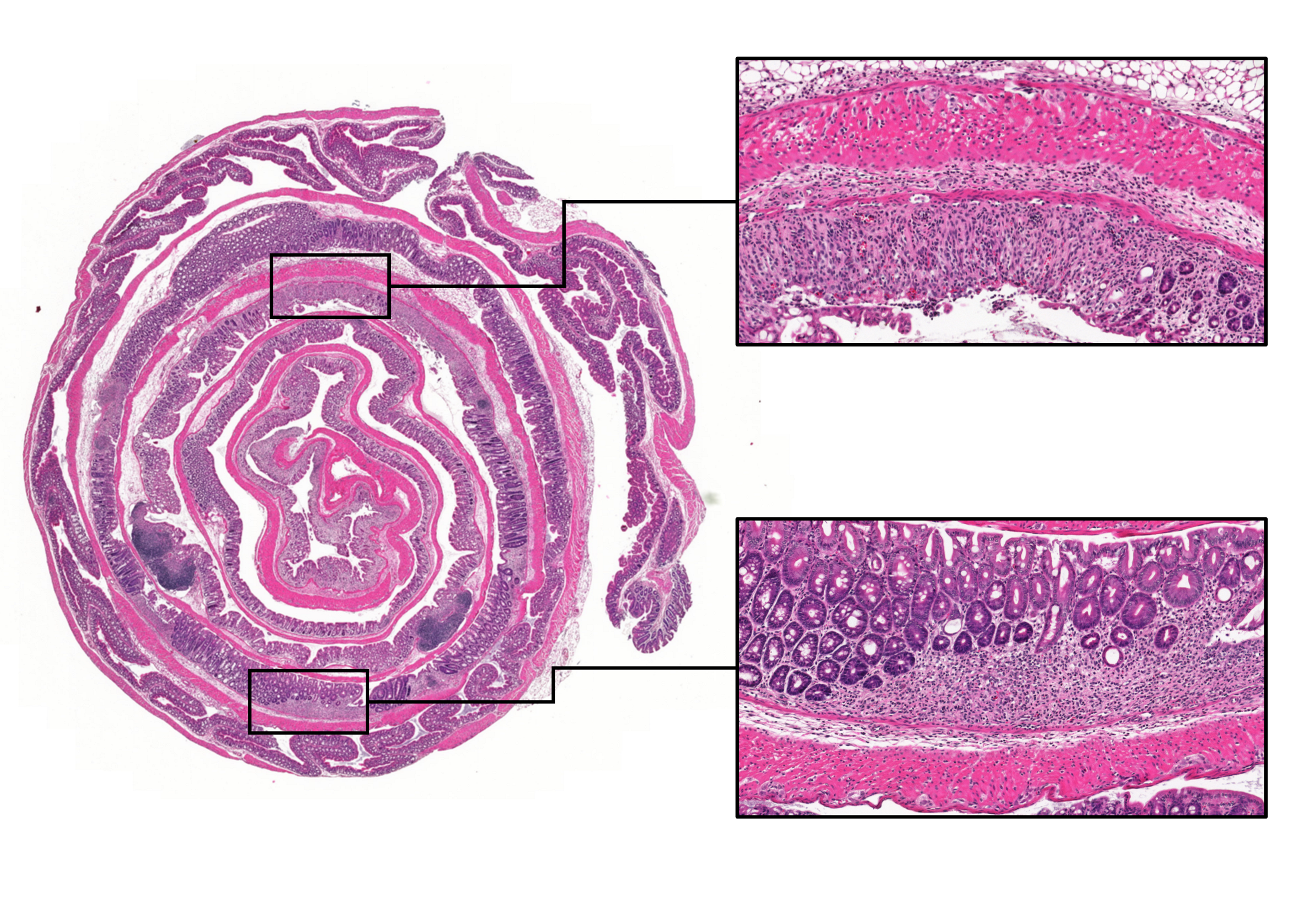


**Supplemental Figure 1. Exemplary whole colon slice after 7 days of DSS administration.** Colonic tissue was harvested, cut lengthwise, rolled, fixed and HE-stained to ensure presence of colitis. Characteristic features of DSS colitis are shown enlarged. DSS colitis is characterized by lymphocytic infiltrates, loss of crypt architecture and mucosal ulcerations.
